# PROTAC technology for sensitizing melanoma to immune checkpoint therapy

**DOI:** 10.1186/s13046-026-03799-z

**Published:** 2026-08-03

**Authors:** Qiujun Zhou, Xiaowei Zhang, Chenlu Zhao, Difei Chen, Chen Chu, Xiaoliang Jin

**Affiliations:** 1 Department of Dermatology, The First Affiliated Hospital, Zhejiang University School of Medicine, Hangzhou, Zhejiang 310003 China; 2 Physical Examination Center, The Fourth Affiliated Hospital of China Medical University, Shenyang, 110032 Liaoning China; 3 Department of First Clinical Medical College, Zhejiang Chinese Medical University, Hangzhou, Zhejiang 310000 China; 4 Department of Clinical Medicine, He University, Shenyang, Liaoning 110163 China; 5 Department of General Surgery, The Fourth Affiliated Hospital of China Medical University, Shenyang, 110032 China; 6 The First Affiliated Hospital of Zhejiang Chinese Medical University (Zhejiang Provincial Hospital of Chinese Medicine), Hangzhou, 310003 China

**Keywords:** PROTACs, Melanoma, Targeted protein degradation, Immunotherapy, Immune checkpoint blockade

## Abstract

Melanoma remains one of the most aggressive malignancies, with limited response to immune checkpoint inhibitors (ICIs) due to primary or acquired resistance. Proteolysis-targeting chimeras (PROTACs) have emerged as a novel therapeutic modality that induces selective degradation of target proteins via the ubiquitin–proteasome system, offering distinct advantages over conventional inhibitors. This review summarizes the design principles and molecular mechanisms of PROTACs, and highlights their emerging role in sensitizing melanoma to immunotherapy. Specifically, PROTACs can restore tumor antigen presentation, reprogram the immunosuppressive tumor microenvironment by targeting regulatory T cells, myeloid-derived suppressor cells, and tumor-associated macrophages, and suppress key oncogenic signaling pathways. We also discuss combination strategies with PD-1/PD-L1 and CTLA-4 blockade, as well as current challenges including poor cell permeability, off-target effects, and acquired resistance. Finally, future perspectives on rational design, tissue-specific delivery systems, and clinical translation are addressed.

## Introduction

Melanoma is one of the most aggressive forms of skin cancer, characterized by high metastatic potential, marked intratumoral heterogeneity, and poor prognosis in advanced stages [1–3]. Although surgical resection is effective for early-stage disease, advanced melanoma remains a major clinical challenge due to rapid disease progression and the emergence of therapeutic resistance [3, 4].

The development of immune checkpoint inhibitors (ICIs) targeting cytotoxic T lymphocyte-associated antigen 4 (CTLA-4) and programmed cell death protein 1 (PD-1)/programmed death ligand 1 (PD-L1) has revolutionized melanoma treatment and significantly improved long-term survival outcomes [5, 6]. Combination strategies such as nivolumab plus ipilimumab have further increased response rates and overall survival, establishing immunotherapy as a cornerstone in melanoma management [7, 8]. However, a substantial proportion of patients either fail to respond (primary resistance) or eventually relapse after an initial response (acquired resistance), limiting the overall clinical benefit of ICIs [9, 10].

Mechanistically, resistance to immune checkpoint blockade is highly complex and involves both tumor-intrinsic and tumor-extrinsic factors. Tumor-intrinsic mechanisms include defects in antigen processing and presentation (e.g., B2M loss), impaired interferon-γ signaling (JAK/STAT pathway alterations), activation of oncogenic signaling pathways, and epigenetic remodeling that suppresses immune recognition [11–13]. Tumor-extrinsic mechanisms are largely driven by an immunosuppressive tumor microenvironment enriched with regulatory T cells, tumor-associated macrophages, and myeloid-derived suppressor cells, which collectively inhibit effective T cell-mediated cytotoxicity [14, 15]. In addition, immune checkpoint redundancy and T cell exhaustion further contribute to immune escape and therapeutic failure [16].

In this context, targeted protein degradation has emerged as an important research direction in the field of cancer therapy. Among these approaches, proteolysis-targeting chimeras (PROTACs) represent a novel class of bifunctional molecules that induce the selective degradation of specific oncogenic or regulatory proteins, thereby shifting the therapeutic paradigm from “functional inhibition” to “protein elimination” [17–19]. Compared with conventional small-molecule inhibitors, PROTACs not only effectively suppress target protein activity but also enable complete removal of proteins, including their non-enzymatic and scaffolding functions, thereby allowing more profound interference with oncogenic signaling networks in cancer cells [20, 21]. Importantly, PROTAC-mediated protein degradation not only affects tumor cell proliferation and survival but may also indirectly regulate antitumor immune responses by reshaping tumor-intrinsic signaling pathways. For instance, sustained degradation of key oncogenic drivers or immune regulatory proteins may enhance tumor antigen presentation, improve type I/II interferon signaling, and attenuate immunosuppressive signaling pathways. Collectively, these effects may increase tumor immunogenicity and ultimately improve tumor sensitivity to immune checkpoint inhibitors [22, 23].

Therefore, from a mechanistic perspective, PROTAC technology is not only a novel antitumor strategy but also a potential bridge linking oncogenic signaling pathway modulation with the enhancement of immunotherapy efficacy, thereby providing new theoretical foundations and strategic directions for overcoming immune checkpoint inhibitor resistance in melanoma. Based on this premise, this review systematically summarizes the emerging roles of PROTACs in tumor immune regulation, with a particular focus on their molecular mechanisms in reshaping the tumor immune microenvironment through targeted protein degradation and enhancing the efficacy of immune checkpoint blockade. In addition, we discuss recent advances in combination strategies involving PROTACs and immunotherapy, and further highlight the translational prospects and current challenges of applying PROTAC-based approaches in melanoma treatment.

## Mechanisms and design of PROTACs

Inside cells, protein degradation is largely governed by the ubiquitin–proteasome system (UPS), a selective mechanism critical for sustaining homeostasis [24]. At the heart of this tightly controlled pathway lie several key players: ubiquitin (Ub) itself, a three-enzyme relay consisting of E1, E2, and E3, the 26 S proteasome, and the target proteins destined for breakdown. The action unfolds in a defined sequence. First, E1 activates ubiquitin and transfers it to E2. Subsequently, an E3 ligase recognizes a specific substrate and facilitates the covalent attachment of ubiquitin to that protein. With repeated rounds of this process, a polyubiquitin chain forms on the substrate. This chain functions as a degradation tag, guiding the tagged protein to the 26 S proteasome, where it is finally fed into the 20 S catalytic core for proteolytic destruction [25].

**Fig. 1:**
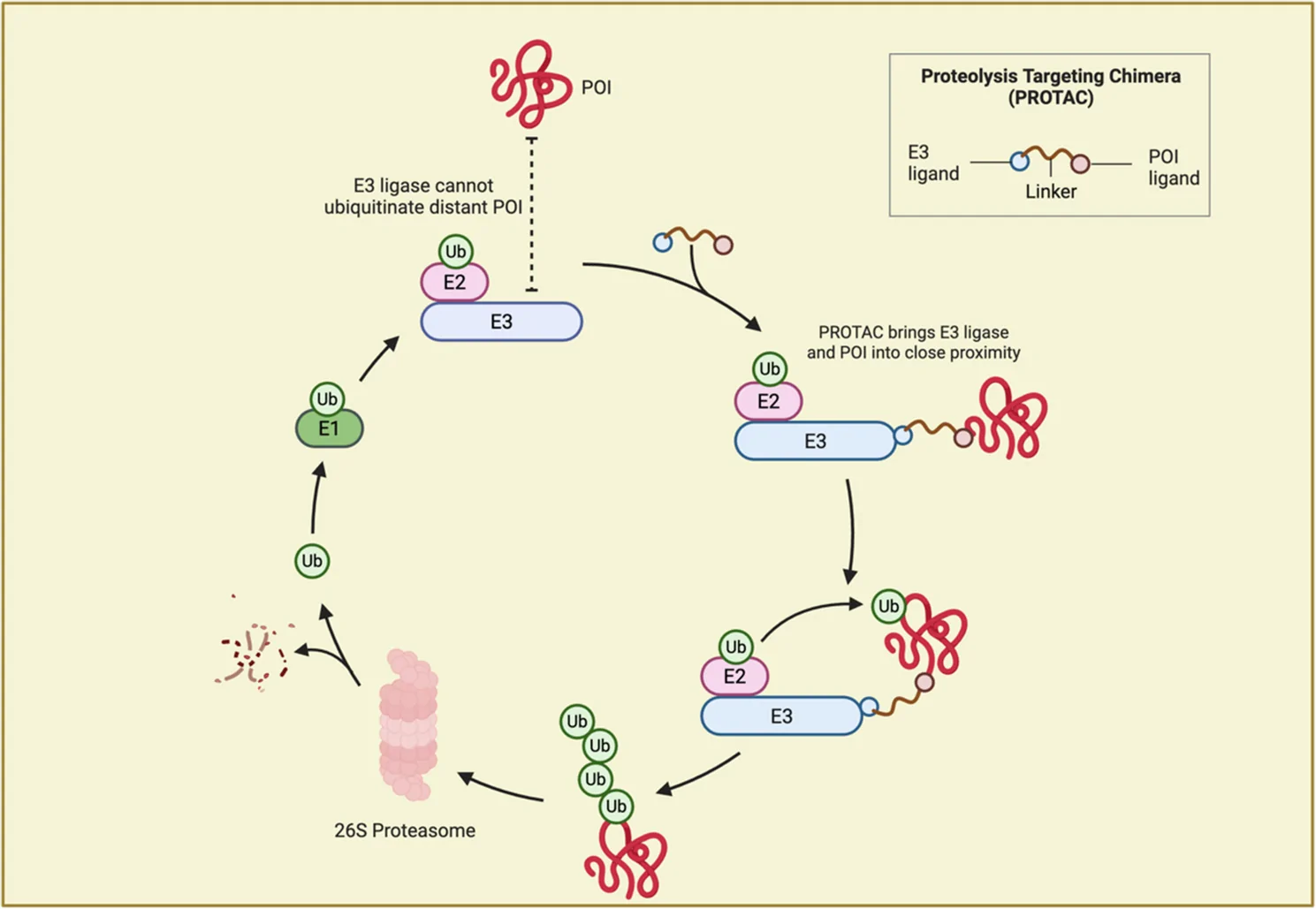
Overview of PROTAC-induced targeted protein degradation. PROTACs are bifunctional molecules consisting of three key components: a ligand that binds the protein of interest (POI), a linker, and a ligand that recruits an E3 ubiquitin ligase. By simultaneously engaging both the POI and an E3 ligase, PROTACs facilitate the formation of a ternary complex, bringing the target protein into close proximity with the ubiquitin-proteasome machinery. This spatial arrangement promotes the transfer of ubiquitin from an E2 conjugating enzyme to the POI, leading to polyubiquitination. The ubiquitin-tagged POI is subsequently recognized and dismantled by the 26 S proteasome. Importantly, PROTACs act through an event-driven rather than occupancy-driven mechanism. Following target ubiquitination, the PROTAC molecule may dissociate and engage additional target molecules, potentially enabling substoichiometric degradation under favorable cellular conditions

Composed of three obligatory elements—a ligand that attaches to the protein of interest (POI), a linker, and a second ligand that recruits an E3 ubiquitin ligase—PROTACs are bifunctional in nature [26]. When administered, each PROTAC molecule binds simultaneously to both the POI and an E3 ligase, giving rise to a transient ternary complex. The spatial proximity established within this complex enables the conjugation of polyubiquitin chains onto the POI. These ubiquitin tags then signal the POI for recognition and eventual breakdown by the proteasome (Fig. 1). A defining pharmacological feature of PROTACs is their event-driven mode of action. Following productive ternary complex formation and target ubiquitination, the PROTAC molecule may dissociate and participate in additional degradation cycles, thereby enabling substoichiometric target depletion under favorable cellular conditions. However, the extent of such molecular recycling is context-dependent and may be limited by cellular efflux, metabolic inactivation, intracellular sequestration, and the availability of functional E3 ligase machinery.

The success of PROTAC-mediated degradation is largely determined by how stable and cooperative the ternary complex proves to be. Within this assembly, non-covalent interactions beyond the intended binding events are often seen—specifically, contacts between the POI (or its ligand) and the E3 ligase, as well as in the reverse direction. These additional interactions enhance the thermodynamic stability and lifespan of the complex [27]. A number of crystal structures depicting such PROTAC-induced ternary configurations have been solved [28, 29], offering essential structural guidance for the rational improvement of PROTAC molecules [30]. To quantitatively describe ternary complex stability, researchers use the cooperativity factor (α), calculated as the ratio between the binary dissociation constants and the ternary complex dissociation constant. An α value greater than 1 denotes positive cooperativity, indicating that the ternary complex is more stable than the combined stability of its binary components [31]. This parameter has been measured using various techniques, including AlphaScreen/AlphaLISA [32], biolayer interferometry [33], surface plasmon resonance [31], and time-resolved fluorescence resonance energy transfer (TR-FRET) [34].

In addition to ternary complex stability, the accessibility and spatial positioning of lysine residues on the POI have been identified as key determinants influencing how effectively degradation proceeds. Evidence from Ciulli and colleagues [35] using cryo-electron microscopy and in vitro ubiquitination assays demonstrated that lysine 456 located on the second bromodomain of BRD4 (Brd4BD2) represents an ideal ubiquitination site for MZ1-triggered degradation. Their results further underscored the importance of geometric compatibility between lysine targets and the E2–Ub catalytic module that works in conjunction with the RING domain of Cullin-RING ligases (CRLs). Lysine residues falling within an optimal distance and exhibiting a favorable orientation relative to this enzymatic center are preferentially modified by ubiquitin. Conversely, those situated in sterically crowded or geometrically suboptimal environments are less susceptible to ubiquitination.

To conclude, the rational design and optimization of next-generation targeted protein degraders rely on a thorough grasp of the entire process underlying PROTAC-induced degradation—from the assembly of the ternary complex through to the ubiquitination of the substrate. This knowledge provides the essential groundwork for advancing the field.

## Mechanisms of PROTAC-Mediated sensitization to immunotherapy in melanoma

In this section, we discuss the distinct mechanisms through which PROTACs can sensitize melanoma to immunotherapy. A comprehensive summary of PROTACs and PROTAC-based strategies that have been directly validated in melanoma models is provided in Table 1.

### Restoration of tumor antigen presentation and immunogenicity

The initiation of tumor-specific immune responses critically depends on the recognition of tumor-associated antigen peptides presented by major histocompatibility complex class I (MHC-I) molecules on the surface of tumor cells by CD8^+^ cytotoxic T lymphocytes. However, melanoma and other solid tumors frequently evade immune surveillance by downregulating antigen processing and presentation machinery, thereby reducing tumor immunogenicity. PROTAC technology provides a novel strategy to restore tumor immunogenicity through the targeted degradation of specific proteins [36, 37].

A landmark study by Massafra and colleagues first demonstrated that PROTACs targeting bromodomain and extra-terminal (BET) proteins can significantly increase the abundance of target-derived peptide antigens presented on MHC-I molecules at the tumor cell surface. This effect was consistently observed across multiple human cell lines derived from both liquid and solid tumors, and was shown to be strictly dependent on E3 ubiquitin ligase recruitment and proteasome-mediated degradation [36]. Mechanistically, upon PROTAC-induced degradation via the ubiquitin–proteasome system, the resulting peptide fragments are efficiently processed and loaded onto MHC-I molecules, which are subsequently transported to the cell membrane for antigen presentation [36]. This finding highlights the potential of PROTACs as “immunogenicity remodelers,” capable not only of eliminating oncogenic proteins but also of enhancing tumor visibility to the immune system. Importantly, the same study further combined PROTAC treatment with T cell–bispecific antibodies and found that PROTAC pretreatment significantly enhanced the expression of T cell activation markers (CD69 and CD25), increased the secretion of granzyme B, interferon-γ (IFN-γ), and tumor necrosis factor-α (TNF-α), and improved tumor cell killing efficiency in co-culture systems [36]. Although the tumor models used in this study were primarily derived from non-melanoma solid tumors such as breast cancer, the underlying mechanism, namely enhanced MHC-I antigen presentation leading to improved T cell-mediated cytotoxicity, is broadly applicable and provides a strong theoretical basis for its potential implementation in melanoma. However, direct experimental validation of this mechanism in melanoma models has not yet been reported, and future studies specifically designed to confirm this PROTAC-mediated immunogenicity enhancement in melanoma are urgently warranted.

**Fig. 2:**
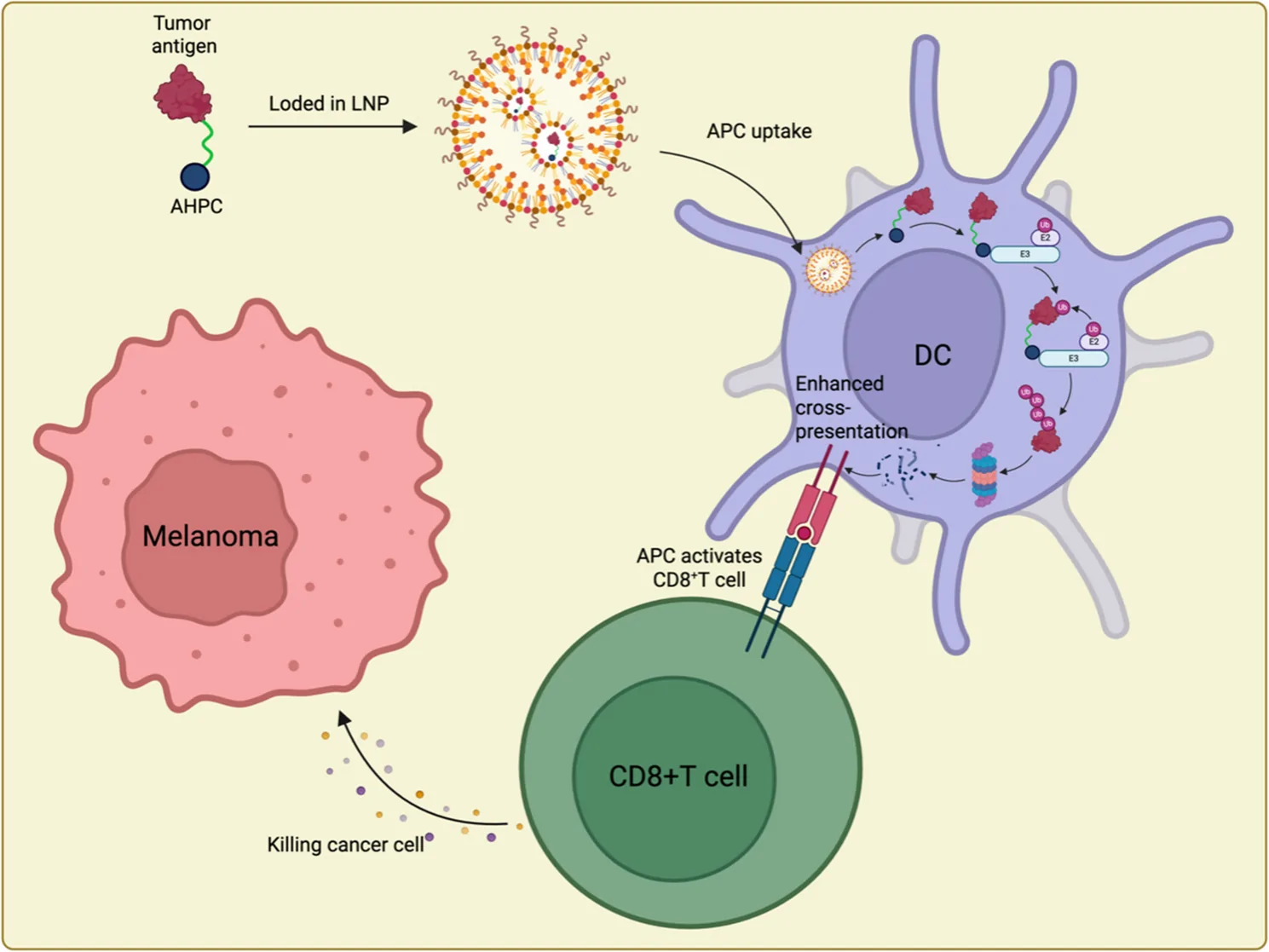
Mechanistic workflow of PROTAC-based vaccine for melanoma immunotherapy. Tumor antigens conjugated with AHPC (E3 ligase recruiter) are loaded into LNPs and delivered to DCs. Within DCs, AHPC recruits endogenous E3 ligases to ubiquitinate the antigens, targeting them for proteasome degradation. The degraded peptide products are cross-presented on MHC-I molecules, triggering robust CD8⁺ T cell activation. These activated CTLs migrate to the tumor site and exert potent cytotoxicity against melanoma cells, effectively inhibiting tumor progression

Extending the PROTAC concept into the field of vaccine development has also yielded remarkable progress. Inspired by PROTAC technology, Xu and colleagues developed an antigen-targeting degradation tumor vaccine (TAgD-TVac) [38]. In this platform, ligands of the E3 ubiquitin ligase (such as AHPC targeting pVHL) are covalently conjugated to tumor antigens and encapsulated within lymph node–targeting lipid nanoparticles. Following subcutaneous administration, the vaccine preferentially accumulates in lymph nodes and is efficiently taken up by antigen-presenting cells (APCs), such as DCs. Within these cells, the conjugated antigen is recruited to endogenous E3 ligases, undergoes ubiquitination, and is subsequently degraded by the 26 S proteasome. The resulting antigen-derived peptides are then cross-presented via MHC-I molecules, thereby effectively activating CD8^+^T cell immune responses [38]. In melanoma mouse models, TAgD-TVac induced robust cytotoxic T cell priming and potent antitumor immune responses. More importantly, this vaccine also elicited strong antigen-specific adaptive immunity and long-term immunological memory across multiple tumor models, including melanoma, triple-negative breast cancer, lung cancer, and ovarian cancer, thereby effectively suppressing tumor growth, metastasis, and recurrence. Notably, combination with immune checkpoint blockade further enhanced therapeutic efficacy [38] (Fig. 2).

Collectively, these studies suggest that PROTAC technology restores tumor antigen presentation and immunogenicity through at least two complementary mechanisms. First, by directly degrading intracellular tumor proteins, PROTACs increase the abundance of protein-derived peptides presented on MHC-I molecules, thereby enhancing tumor immune visibility. Second, as a key design element in vaccine development, PROTAC-inspired strategies promote efficient ubiquitin–proteasome processing of exogenous antigens, thereby enhancing cross-presentation and more effectively activating CD8^+^T cell responses. Together, these mechanisms provide a strong molecular basis for improving melanoma sensitivity to immunotherapy.

### Reprogramming of the tumor immune microenvironment

**Fig. 3:**
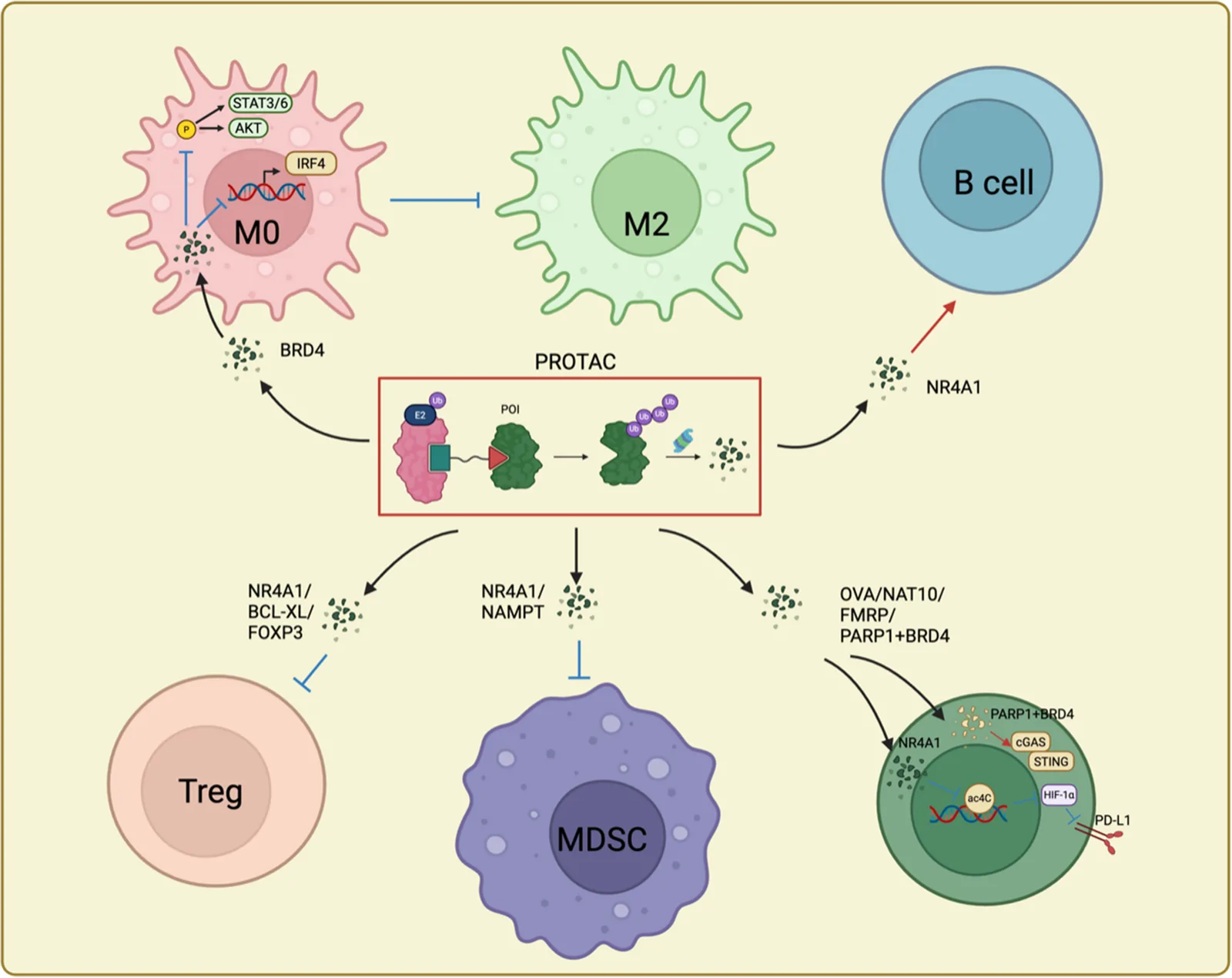
PROTAC-mediated reprogramming of the tumor immune microenvironment in melanoma. PROTACs target multiple immunosuppressive cell populations to enhance antitumor immunity. In TAMs, BRD4 degradation suppresses M2 polarization via IRF4/STAT3/AKT inhibition. In MDSCs, degradation of NR4A1 or NAMPT relieves CD8⁺ T cell suppression. In Tregs, targeting NR4A1, BCL-XL, or FOXP3 reduces their abundance and suppressive function. NR-V04 also promotes B cell accumulation. In CD8⁺ T cells, PROTAC-based strategies (PROTAV, NP1192, sc1-VHLL, PROP + B) enhance activation, reduce exhaustion, and activate the cGAS-STING pathway. These coordinated effects reshape the TIME and sensitize melanoma to immunotherapy

Beyond restoring antigen presentation, PROTACs can also remodel the immunosuppressive tumor microenvironment (TIME) by targeting key immune cell subsets that dampen antitumor immunity (Fig. 3). Regulatory T cells (Tregs) are a central cellular component responsible for maintaining the TIME in melanoma [39]. Tregs are highly enriched in melanoma tissues, and their abundance is closely associated with poor patient prognosis and resistance to immune checkpoint blockade therapy [40, 41]. Single-cell transcriptomic studies have shown that NR4A1 is a key marker of TCR activation in Tregs and is essential for maintaining Treg lineage identity [42]. Based on this observation, a PROTAC molecule targeting NR4A1 (NR-V04) was developed. In melanoma model, NR-V04 treatment significantly reduced tumor-infiltrating Treg levels, thereby effectively reversing the local immunosuppressive state. Mechanistically, NR-V04 induces selective degradation of NR4A1, thereby disrupting the transcriptional regulatory network that maintains Treg identity and suppressive function [43]. This leads to attenuation of the immunosuppressive program and reduced functional stability of Tregs within the TME, highlighting the ability of PROTACs to directly reprogram Treg functional states by targeting lineage-defining transcriptional regulators. Beyond NR4A1, BCL-XL represents another critical pro-survival dependency factor in tumor-infiltrating Tregs. Studies have demonstrated that BCL-XL is consistently upregulated in tumor-infiltrating Tregs across multiple solid tumors, including melanoma, whereas its expression remains relatively low in peripheral Tregs, suggesting a tumor-associated survival advantage [44]. Based on this characteristic, a platelet-sparing, BCL-XL–selective PROTAC (DT2216) was developed, which induces selective degradation of BCL-XL via recruitment of the VHL E3 ubiquitin ligase [45]. In solid tumor models, DT2216 treatment significantly reduced tumor-infiltrating Treg abundance and enhanced intratumoral CD8^+^T cell activity, without inducing detectable platelet toxicity, indicating favorable selectivity and safety. These findings suggest that PROTACs can modulate Treg biology not only by disrupting transcriptional programs but also by targeting key survival dependencies, thereby reshaping the immunosuppressive TME [45]. Although most evidence derives from non-melanoma models, the similar expression pattern of BCL-XL in melanoma-infiltrating Tregs supports its potential translational relevance in melanoma immunotherapy. In addition, PROTAC strategies targeting the lineage-defining transcription factor FOXP3 further extend the potential for direct Treg modulation. A PROTAC molecule derived from an FOXP3 inhibitory peptide (P60-L3-VHL) has been designed to induce FOXP3 degradation via the ubiquitin–proteasome system, thereby suppressing Treg differentiation and FOXP3-dependent gene expression programs. In vitro results demonstrate reduced FOXP3 levels and impaired Treg generation following treatment [46]. However, this strategy remains at the cellular validation stage, and its in vivo efficacy within the melanoma TIME, as well as its impact on antitumor immunity, requires further investigation.

Myeloid-derived suppressor cells (MDSCs) represent another important immunosuppressive cell population within the tumor immune microenvironment. MDSCs suppress T cell function through various mechanisms, including the production of arginase, inducible nitric oxide synthase, and reactive oxygen species, thereby promoting tumor progression and treatment resistance [47, 48]. In melanoma patients, the accumulation of MDSCs is closely associated with resistance to immunotherapy [49]. Therefore, targeting MDSCs has emerged as an important strategy to overcome immunotherapy resistance. The NR4A1-targeting PROTAC molecule NR-V04 has demonstrated significant efficacy in modulating MDSCs. Flow cytometry analysis revealed that in B16F10 melanoma-bearing mice, NR-V04 treatment significantly reduced the percentage of monocytic MDSCs in both tumor tissues and blood, whereas MDSC levels in the spleen remained largely unaffected. This tissue-specific effect indicates that NR-V04 primarily targets MDSCs within the TME rather than systemic MDSCs [50]. Mechanistically, NR4A1 is highly expressed in MDSCs, and its degradation alleviates MDSC-mediated suppression of CD8⁺T cells, thereby unleashing antitumor immune responses [50]. Beyond melanoma models, studies in other tumor types have also provided mechanistic evidence for PROTAC-mediated modulation of MDSCs. Wu and Sheng’s team discovered in colorectal cancer models that NAMPT promotes the expansion of tumor-infiltrating MDSCs through its non-enzymatic activity [51]. It should be noted, however, that the NAMPT-targeting PROTAC A7 has been validated exclusively in colorectal cancer models, and direct experimental evidence for its efficacy in melanoma models remains lacking. Based on this observation, the team developed PROTAC A7, the first NAMPT-targeting PROTAC molecule, which significantly inhibited tumor-infiltrating MDSCs and enhanced antitumor immune responses by degrading NAMPT [51]. Although this study was primarily conducted in colorectal cancer models, NAMPT is also highly expressed in melanoma [52] and is associated with immunosuppression [53]. Therefore, the NAMPT-targeting PROTAC strategy holds clear translational potential for the modulation of MDSCs in melanoma.

Tumor-associated macrophages (TAMs) also play a critical immunosuppressive role in the melanoma TIME, primarily exhibiting an M2-polarized phenotype that promotes tumor progression and immune evasion [54, 55]. Studies have shown that BRD4 plays an important role in maintaining the M2 polarization state of TAMs. In one study, a BRD4 PROTAC (ARV825) was co-delivered with an oxygen-generating microneedle patch for localized precision treatment of postoperative melanoma. This strategy reduced M2-type TAM infiltration and suppressed PD-L1 expression through BRD4 degradation, which synergized with oxygen supply to remodel the immunosuppressive melanoma microenvironment, thereby enhancing the antitumor activity of cytotoxic T lymphocytes [56]. Mechanistically, BRD4 degradation suppresses M2 macrophage polarization by inhibiting IRF4 promoter transcription as well as STAT6, STAT3, and AKT phosphorylation. Although this mechanism was primarily elucidated in glioma models, the molecular pathways through which BRD4 regulates TAM function are likely conserved across different tumor types; however, dedicated experiments specifically designed to validate BRD4-mediated TAM modulation in the melanoma microenvironment are still required to confirm its translational relevance in this malignancy [57]. Currently, studies on PROTAC-mediated direct regulation of TAM polarization in melanoma remain very limited. Future development of PROTAC strategies targeting key regulatory molecules such as STAT3 and CSF-1R represents an important direction in this field.

Furthermore, the role of B cells in the melanoma TIME is gaining increasing attention. Studies have shown that NR-V04 treatment in B16F10 melanoma-bearing mice leads to significant accumulation of tumor-infiltrating B cells, and single-cell RNA sequencing analysis revealed that NR4A1 is highly expressed in B cells within the melanoma TIME. Importantly, in B cell-deficient mice inoculated with B16F10 tumors, NR-V04 completely lost its antitumor efficacy, confirming that B cell accumulation is essential for the antitumor immune effects of NR-V04 [50]. Mechanistically, NR4A1 functions as a negative feedback regulator in B cells: under physiological conditions, NR4A1 restricts B cell expansion in response to antigen stimulation and suppresses the survival of self-reactive B cells in peripheral tissues; NR-V04 relieves this negative feedback by degrading NR4A1, thereby promoting B cell proliferation [50]. Of note, the modulatory effect of NR-V04 on B cells has been observed not only in the B16F10 model but also in the Yummer 1.7 melanoma model carrying clinically relevant human melanoma-associated driver mutations, suggesting the generalizability of this mechanism. To date, this is the only report of PROTAC-mediated direct modulation of B cells in melanoma, highlighting that targeting B cell immune regulation may represent a potential new direction for PROTAC drug design.

CD8^+^ cytotoxic T lymphocytes are the core effector cells of antitumor immunity, and their tumor infiltration density and functional status are critical determinants of immunotherapy success. PROTAC technology enhances the recruitment and function of CD8^+^T cells through multiple mechanisms, with direct validation in melanoma and other tumor models. Recently, a pH-responsive PROTAC-based nanosystem (MNC@Ca/MnCO₃/ARV/anti-PD1) was developed for in situ tumor vaccination in melanoma [58]. This system delivers the BRD4 PROTAC ARV-825 to induce tumor-specific ferroptosis, which releases tumor-associated antigens while simultaneously capturing these antigens for enhanced presentation by APCs. In the B16F10 melanoma model, this nanosystem significantly increased intratumoral CD8⁺ T cell infiltration and activation, and demonstrated potent antitumor efficacy when combined with anti-PD1 therapy through remodeling of the immunosuppressive tumor microenvironment [58]. However, it should be noted that PROTAC-induced antigen release is inherently transient—since the target protein is rapidly degraded, the supply of antigenic peptides may quickly decline, which could limit the persistence of CD8⁺ T cell activation [22]. This suggests that, unlike conventional PROTAC optimization pursuing rapid degradation, designing PROTACs with slower degradation kinetics may be necessary to maintain sustained antigen presentation and T cell stimulation [22]. Targeting metabolic regulators to enhance CD8⁺ T cell function represents another important strategy. The NAT10-targeting PROTAC NP1192 eliminates ac4C modification on HIF1A mRNA by degrading NAT10, thereby inhibiting HIF-1α-mediated lactate production and PD-L1 upregulation [59]. Single-cell RNA sequencing and in vitro co-culture experiments demonstrated that NP1192 combined with anti-PD-L1 treatment significantly expanded IFN-γ^+^ effector CD8^+^T cells while reducing the proportion of exhausted CD8^+^T cells [59]. Although the NP1192 PROTAC was evaluated exclusively in a cervical cancer model, hypoxia and glycolytic reprogramming are universal features of the melanoma TIME, giving this strategy clear translational potential; nevertheless, its ability to modulate CD8⁺ T cell function within the melanoma immune microenvironment awaits direct experimental confirmation. PROTACs targeting RNA-binding proteins have also shown promise. The G-quadruplex RNA-based PROTAC sc1-VHLL achieves specific degradation of the RNA-binding protein FMRP. In the CT26 colorectal cancer model, FMRP degradation significantly promoted intratumoral infiltration of lymphocytes and CD8^+^T cells while reducing the proportion of Tregs, converting a “cold” tumor into a “hot” tumor [60]. Notably, FMRP was previously considered an “undruggable” target, and this study provides proof-of-concept that PROTAC technology can target RNA-binding proteins for cancer immunotherapy; however, the therapeutic potential of sc1-VHLL in melanoma has not yet been experimentally demonstrated. Dual-targeting degradation strategies enhance CD8⁺ T cell responses by activating innate immune pathways. The PROP + B strategy simultaneously degrades PARP1 and BRD4, inducing DNA damage and cytosolic dsDNA accumulation, thereby activating the cGAS-STING pathway. In the B16F10 melanoma model, this strategy directly induced robust CD8^+^T cell and NK cell-mediated antitumor immune responses and suppressed lung metastasis progression [61]. Mechanistically, PARP1 degradation disrupts base excision repair, while BRD4 degradation epigenetically silences core components of homologous recombination, leading to the collapse of DNA repair systems and driving nuclear DNA leakage into the cytosol, which subsequently activates the STING pathway [61]. However, the clinical safety of such multi-target degradation strategies remains unclear—systemic administration may simultaneously affect the same targets in both CD8⁺ T cells and Tregs, leading to counterproductive effects or excessive immune activation and autoimmune toxicity, which will be a critical issue for clinical translation. Currently, evidence for PROTAC-mediated direct enhancement of CD8^+^T cell function is primarily derived from indirect mechanisms (improved antigen presentation, relief of metabolic suppression, activation of STING pathways), whereas PROTAC strategies directly targeting the endogenous exhaustion regulatory network within CD8^+^T cells remain at an early exploratory stage. Transcription factors often exert pleiotropic effects in T cell differentiation, and complete degradation may lead to unexpected biological consequences. For example, TOX is a core transcription factor that maintains CD8^+^T cell exhaustion, yet TOX knockout impairs CD8^+^T cell effector function and persistence, even though it prevents the expression of exhaustion-associated genes [62]. This paradox suggests that intervention in the T cell transcriptional regulatory network requires more sophisticated design. It has been suggested that future PROTAC designs for targeting transcription factors should pursue partial degradation or selective degradation of specific protein-protein interaction interfaces rather than complete elimination of target protein function [62]. Furthermore, the development of delivery systems with tumor microenvironment or CD8^+^T cell specificity (such as nanocarriers or prodrug strategies) represents a key direction for achieving precise regulation.

### Suppression of immunosuppressive signaling pathways

Melanoma cells evade immune clearance through sustained activation of multiple immunosuppressive signaling pathways, among which abnormal activation of STAT3, NF-κB, PI3K/AKT, and WNT/β-catenin pathways is particularly prominent. PROTACs can suppress immunosuppressive signal transduction at the source by targeting and degrading core driver proteins within these pathways.

STAT3 is a critical transcription factor linking inflammation, immunosuppression, and tumor progression. In multiple solid tumors including melanoma, persistent activation of STAT3 upregulates immunosuppressive molecules such as PD-L1, IL-10, and VEGF while inhibiting dendritic cell maturation and T cell recruitment. The dual-targeting NP-PROTACs developed by Lu and colleagues effectively degraded both STAT3 and β-catenin simultaneously, blocking reciprocal feedback activation between these pathways [45]. Following NP-PROTAC treatment, the levels of immunosuppressive cytokines in tumor tissues significantly decreased, while CD103⁺ DC infiltration increased, further promoting T cell activation and tumor killing [45]. Given the strong association between WNT/β-catenin pathway activation in BRAF-mutant melanoma and primary resistance to immunotherapy [63], PROTAC-based targeting of β-catenin has clear translational potential.

In addition, aberrant activation of the NF-κB signaling pathway also contributes to the formation of the immunosuppressive microenvironment in melanoma. In B16F10 mouse melanoma cells, LD4172, a PROTAC degrader targeting RIPK1, efficiently and specifically degrades RIPK1, effectively inhibits TNF-induced canonical NF-κB signaling, and simultaneously induces tumor cell apoptosis and immunogenic cell death. In in vivo studies, in B16F10 melanoma-bearing mouse models, LD4172 achieves tumor-selective RIPK1 degradation and synergizes with anti-PD-1 therapy, significantly reducing tumor weight, prolonging survival, and remodeling the tumor immune microenvironment [64]. Therefore, LD4172 serves as a valuable chemical probe for studying NF-κB signaling function and RIPK1-targeted immunomodulation in melanoma.

Based on the findings discussed above, additional tumor-intrinsic immune evasion pathways may represent promising contexts for PROTAC application in melanoma. PTPN2 functions as a critical negative regulator of antitumor immunity by dephosphorylating JAK-STAT and TCR signaling, and its deficiency enhances response to immune checkpoint blockade [65]; PTPN2-targeting PROTACs have recently been developed [66], though their evaluation in melanoma remains unexplored. TBK1 represents another compelling target, with a selective PROTAC degrader demonstrating improved selectivity and in vivo antitumor activity [67]. RNF31, a component of the linear ubiquitin chain assembly complex, has been implicated in immune evasion through p53 regulation [68], and while no RNF31-targeting PROTAC has been reported to date, its E3 ligase and scaffolding functions make it an attractive candidate for degrader-based strategies. These examples suggest that PROTAC technology may have broader applicability in targeting diverse tumor-intrinsic immune evasion pathways beyond those already investigated, warranting further exploration in melanoma.

Recent studies have demonstrated that PROTAC technology can also restore anti-tumor immunity by directly targeting immune checkpoint molecules and related immunosuppressive enzymes. For example, Liu et al. developed PD-L1/SHP2 dual-targeting PROTACs (BS-7 C-V2 and BS327V2), which efficiently degrade both target proteins in A375 and B16-F10 melanoma cells and demonstrate significant antitumor efficacy in B16-F10 tumor-bearing mouse models [69]. In addition, Rong et al. designed a dual-targeting fluorinated peptide PROTAC (DFP-PROTAC) capable of simultaneously degrading PD-L1 and Bcl-xL, synergistically restoring antitumor immunity and enhancing apoptosis sensitivity in the B16-F10 melanoma model [70]. While these findings are encouraging, the long-term safety and off-tumor toxicity of dual-targeting degradation remain to be fully evaluated, and future studies utilizing physiologically relevant melanoma models and comprehensive toxicological profiling are warranted to advance these PROTACs toward clinical translation.

**Table 1:** PROTACs validated in melanoma models

Target Protein	Melanoma Model	Key Effects in Melanoma	ICI Combination Status	Reference
NR4A1	B16F10, Yummer 1.7	Reduces Tregs and MDSCs, promotes B cell accumulation, and induces antitumor efficacy	Not yet combined	[43, 50]
Tumor antigens	B16F10	Induces antigen cross-presentation, activates CD8⁺ T cells, and establishes long-term immunological memory	Anti-PD-1 and anti-CTLA-4 combination showed enhanced efficacy	[38]
BRD4		Reduces M2-type TAM infiltration, suppresses PD-L1 expression, and remodels the immunosuppressive TME	Anti-PD-1 combination potential	[56]
BRD4	B16F10	Increases CD8⁺ T cells	Anti-PD-1 combination showed enhanced efficacy	[58]
PARP1 + BRD4	B16F10	Activates cGAS-STING pathway, induces CD8⁺ T and NK cell responses, and suppresses lung metastasis	Not reported	[61]
RIPK1	B16F10	Inhibits NF-κB signaling, induces apoptosis and immunogenic cell death, and remodels TME	Anti-PD-1 combination showed enhanced efficacy	[64]
PD-L1 + SHP2	A375, B16F10	Degrades PD-L1 and SHP2, enhances T cell killing, and shows in vivo antitumor activity	Anti-PD-1 combination showed enhanced efficacy	[69]
PD-L1 + Bcl-xL	B16F10	Degrades PD-L1 and Bcl-xL, restores antitumor immunity, and enhances apoptosis sensitivity	Anti-PD-1 combination showed enhanced efficacy	[70]

## Combination strategies of PROTACs with immunotherapy

Given that PROTACs are unlikely to function as monotherapies in advanced melanoma, defining optimal combination regimens with established immunotherapies is a priority for translational research.

### PROTAC Combination with D-1/PD-L1 blockade

The combination of PD-L1-targeting PROTACs with anti-PD-1/PD-L1 antibodies represents an important strategy for sensitizing melanoma to immune checkpoint inhibitor therapy. The mechanistic rationale for this combination lies in the complementary actions of the two modalities: PROTAC-mediated degradation of PD-L1 on tumor cells removes the immunosuppressive signal at its source, while supplemental antibody blockade can “cover” residual PD-L1-expressing cells that may escape degradation, thereby ensuring more comprehensive inhibition of the PD-1/PD-L1 axis [22].

Several preclinical studies have provided evidence supporting this combination strategy. Liu and colleagues demonstrated that the PD-L1-targeting PROTAC BMS-37-C3 significantly reduced PD-L1 protein levels in A375 and B16-F10 melanoma cells. In co-culture experiments of T cells with A375 cells, BMS-37-C3 effectively enhanced T cell-mediated tumor cell killing. Flow cytometry analysis further confirmed that BMS-37-C3 efficiently reduced cell surface PD-L1 expression levels [71]. In subsequent work, the same team designed the first dual-targeting PROTAC molecules, BS-7 C-V2 and BS327V2, which simultaneously degrade PD-L1 and SHP2 [69]. SHP2 is a key phosphatase downstream of the PD-1 signaling pathway that mediates T cell functional inhibition. These dual-targeting PROTACs exhibited significant in vivo antitumor activity in B16-F10 melanoma xenograft models, with the mechanism involving enhancement of T cell killing capacity [69]. Notably, even with this dual intervention simultaneously targeting two nodes of the PD-1 pathway, combination with anti-PD-1 antibody still showed enhanced efficacy, suggesting that the complexity of immune checkpoint pathways may allow additive or synergistic effects from multi-level intervention strategies [69]. This additional benefit may reflect the complementary mechanisms of the two approaches. PROTAC-mediated degradation reduces intracellular and cell-surface PD-L1 and may limit its replenishment, whereas anti-PD-1 antibodies can maintain checkpoint blockade when degradation is incomplete or heterogeneous. Moreover, anti-PD-1 therapy can block PD-1 signaling mediated by both PD-L1 and PD-L2, while PD-L1-targeting degradation does not directly affect PD-L2. However, the optimal dosing schedule for this combination remains unexplored. It is currently unclear whether concurrent administration or sequential treatment, such as PROTAC pretreatment followed by anti-PD-1 therapy, would provide greater therapeutic benefit.

The PROTAV vaccine strategy has also been explored in combination with immune checkpoint blockade. In the B16F10 melanoma model, combination therapy with PROTAV-TgT and anti-PD-1 antibody significantly inhibited tumor growth and prolonged mouse survival. In the BRAF V600E-mutant SM1 melanoma model, the triple combination of PROTAV-TgT with anti-PD-1 and anti-CTLA-4 antibodies also demonstrated robust antitumor activity [72].

It should be noted that although these preclinical findings are encouraging, research on PROTAC and ICI combination strategies in melanoma therapy remains at an early stage. Current challenges in this field include the fact that PD-L1, as a transmembrane protein, is primarily degraded through the lysosomal pathway rather than the classical ubiquitin-proteasome pathway, which poses special requirements for conventional PROTAC design. Researchers are developing novel lysosome-based inducible proximity strategies to address this challenge [73]. These preclinical findings establish a theoretical foundation for further development of PROTAC and ICI combination regimens, though significant progress remains before clinical translation.

### PROTAC Combination with CTLA-4 Blockade

CTLA-4 blockade with ipilimumab has been largely supplanted by anti-PD-1 antibodies in front-line melanoma therapy due to its higher toxicity profile, yet the combination of PD-1 and CTLA-4 blockade (nivolumab plus ipilimumab) remains a highly effective regimen for select patient populations [74]. The primary limitation of CTLA-4 antibodies has been their severe immune-related adverse events (irAEs), which reach grade 3–4 in up to 60% of patients at therapeutic doses. These toxicities manifest as rash, hepatotoxicity, cytokine storm, colitis, and cardiovascular alterations, significantly restricting the clinical application of CTLA-4 blockade [74].

Given these considerations, the potential of PROTACs to enhance CTLA-4 blockade efficacy warrants investigation through two complementary mechanisms. First, PROTACs that degrade proteins involved in T cell priming—such as NR4A1, a nuclear receptor transcription factor that limits early T cell activation—could potentially enhance the efficacy of CTLA-4 blockade [75, 76]. Studies have shown that NR4A1 is induced upon chronic antigen stimulation and promotes T cell exhaustion by binding to and regulating exhaustion-associated genes including PD-1, TIM-3, and LAG-3 [75]. CTLA-4 functions primarily in lymph nodes during the initial priming phase of T cell activation, where it competes with CD28 for binding to CD80/CD86 on antigen-presenting cells. Degrading negative regulators of T cell priming at this stage could lower the threshold for T cell activation and synergize with CTLA-4 inhibition. Second, given that CTLA-4 is highly and stably expressed on Tregs within the tumor microenvironment [77, 78], PROTAC-mediated degradation of CTLA-4 itself on intratumoral Tregs could enhance Treg depletion and reverse immunosuppression. Research has demonstrated that Foxp3 + Tregs constitutively express high levels of CTLA-4, which removes CD80/CD86 from antigen-presenting cells via trans-endocytosis, thereby inhibiting effector T cell activation [77]. This approach might offer advantages over antibody-mediated CTLA-4 blockade by eliminating the protein entirely rather than merely blocking its ligand-binding function, potentially achieving more sustained and complete inhibition of Treg-mediated immunosuppression. However, it must be noted that CTLA-4-targeting PROTACs have not yet been reported in the literature, and this remains a theoretical strategy requiring experimental validation.

Despite this conceptual promise, direct evidence for PROTAC + anti-CTLA-4 combinations in melanoma is currently lacking. Most PROTACs evaluated to date have been studied primarily within the tumor microenvironment rather than in secondary lymphoid organs where T cell priming occurs. This represents a critical underexplored area warranting investigation. Future research should consider developing PROTACs that target CTLA-4 itself or key negative regulators of T cell priming, optimizing delivery to lymph nodes, and evaluating combination regimens with existing CTLA-4 antibodies in preclinical melanoma models.

## Current limitations and strategic challenges in PROTAC-based melanoma therapy

Despite the substantial advances and growing therapeutic potential of PROTACs in oncology, several obstacles continue to restrict their broader clinical application [79]. Major challenges involve poor membrane penetration due to their relatively large molecular structures, possible toxicity arising from unintended distribution in normal tissues, inadequate target specificity that may trigger undesirable off-target activities, and the development of acquired resistance during treatment. This section discusses these limitations in detail and summarizes emerging strategies in PROTAC engineering designed to address these concerns. A better understanding and optimization of these aspects may contribute to improving the safety profile, therapeutic efficacy, and translational potential of PROTAC-based interventions.

### Limited cell permeability

Efficient intracellular delivery is a critical determinant of PROTAC functionality, as these molecules must traverse cellular membranes to engage their targets. Nevertheless, the relatively large size of many PROTACs often places them beyond the conventional limits of the “Rule of 5” (molecular weight < 500 Da), which is commonly used to predict the pharmacokinetic properties of orally active small-molecule drugs. Interestingly, despite violating these classical criteria, numerous PROTAC compounds still exhibit potent intracellular degradation activity. This phenomenon may be attributed to their event-driven pharmacological mechanism, in which a single PROTAC molecule can repeatedly mediate the degradation of multiple target proteins, reducing the requirement for high intracellular concentrations. Furthermore, optimization of linker design may facilitate the concealment of polar functional groups and reduce exposed polar surface area, thereby enhancing membrane permeability and improving cellular uptake [80].

To overcome the poor membrane permeability associated with many PROTAC molecules, multiple optimization strategies have been investigated. A widely adopted method focuses on rational linker engineering, such as decreasing overall polarity, reducing hydrogen bond donors, or replacing polar moieties with more hydrophobic components to facilitate membrane diffusion. In parallel with these structural refinements, several unconventional delivery strategies have also been proposed. For example, Lebraud et al. [81] introduced a bioorthogonal intracellular assembly approach in which two individually permeable precursor molecules—a tetrazine-linked thalidomide derivative and a trans-cyclooctene (TCO)-modified JQ1 ligand—enter cells separately and subsequently react intracellularly through click chemistry to form an active BRD4-directed PROTAC. This in situ assembly strategy effectively circumvents the intrinsic permeability challenges associated with large bifunctional molecules. Moreover, cellular uptake is also strongly affected by physicochemical characteristics, including lipophilicity and water solubility. To further improve intracellular transport efficiency, several delivery platforms have been explored, such as nanoparticle-based systems, aptamer-mediated delivery, and cell-penetrating peptide-assisted transport [82]. As an example, Chen et al. [83] demonstrated that loading a pre-assembled ARV-771 construct into lipid-like nanoparticle formulations markedly enhanced cellular uptake efficiency, which consequently resulted in improved target protein degradation activity.

In the context of melanoma, these intracellular and intratumoral delivery barriers pose equally significant challenges and represent critical obstacles that must be addressed for successful clinical translation. The melanoma TME harbors multiple physical and biological barriers that severely restrict the effective delivery of macromolecular therapeutics such as PROTACs. First, the skin tissue architecture of primary melanoma lesions itself constitutes a major anatomical barrier. The stratum corneum, as the outermost layer of the skin, is composed of multiple layers of dead corneocytes tightly packed with abundant intercellular lipids, representing the primary rate-limiting step for transdermal drug delivery [84]. Conventional topical administration struggles to penetrate this dense structure, and PROTAC molecules, typically possessing molecular weights exceeding 800 Da along with specific physicochemical properties, exhibit even lower efficiency in traversing the stratum corneum [85]. To overcome this obstacle, researchers have developed various strategies, including the use of lipid nanocarriers such as highly deformable transfersomes, which can squeeze through the intercellular spaces of the stratum corneum to deliver drugs to deeper skin layers [85]. Additionally, microneedle technology, as an emerging physical permeation enhancement approach, can directly create microchannel pores on the skin surface, bypassing the stratum corneum barrier and enabling direct intratumoral delivery of PROTAC molecules [86]. Second, once PROTACs enter the melanoma tissue, their further diffusion and distribution remain hindered by physical barriers posed by the TME. Abundant activated cancer-associated fibroblasts (CAFs) are present in melanoma tissues, which continuously secrete extracellular matrix (ECM) components including collagen, fibronectin, and hyaluronic acid, forming a dense fibrotic matrix network [87]. This increased matrix density and tissue stiffness not only directly impede the diffusion of PROTAC molecules into the tumor parenchyma but also further restrict convective transport and drug penetration through elevated interstitial fluid pressure (IFP) [84]. Clinical studies have demonstrated that IFP is significantly elevated in melanoma lesions, with non-responsive patients exhibiting baseline IFP levels of 24.4 mmHg that further increase following treatment, whereas responsive patients show markedly reduced IFP, indicating that high IFP directly limits the effective distribution of therapeutic agents within primary melanoma lesions [88]. To address the ECM barrier, ECM-degrading enzymes such as hyaluronidase and collagenase have been employed to reduce tissue density and promote drug penetration [89, 90]. Hyaluronidase degrades hyaluronic acid, a major ECM component in melanoma, lowering interstitial fluid pressure and enhancing nanoparticle accumulation and penetration, as demonstrated in melanoma models. Collagenase treatment has also been shown to increase drug diffusion by 2- to 10-fold in B16F10 melanoma, though its clinical translation is limited by potential toxicity to healthy tissues. These ECM-modulating strategies offer complementary approaches to overcoming physical barriers in melanoma.

### Off-tissue toxicity

Despite their promising therapeutic advantages, PROTACs still face a critical safety limitation related to insufficient tissue selectivity. In many cases, these molecules are unable to fully discriminate between tumor and normal cells, resulting in unintended degradation of target proteins in healthy tissues and thereby inducing off-target toxic effects. This issue becomes especially significant when the protein of interest is indispensable for normal cellular physiology. A representative example is ARV-110, a clinically investigated PROTAC for prostate cancer, which has demonstrated encouraging antitumor activity while simultaneously highlighting the necessity of improving tissue specificity to reduce collateral damage to normal organs [91].

To improve tissue specificity in melanoma, a rational approach is to exploit distinctive pathological features of the melanoma microenvironment rather than relying on generalized delivery strategies that may not apply to this malignancy. Melanoma lesions often exhibit elevated levels of reactive oxygen species (ROS), acidic extracellular pH, and upregulated expression of certain proteases [92], all of which provide exploitable cues for engineering conditionally activated PROTAC systems. By incorporating tumor microenvironment–responsive linkers or protective masking elements, PROTAC molecules can be designed to remain pharmacologically silent in physiological conditions and become activated only upon exposure to melanoma-specific stimuli, thereby improving spatial selectivity and reducing off-target effects [93–95]. For instance, ROS-responsive PROTAC platforms have been reported to preferentially induce target protein degradation in oxidative environments, aligning well with the elevated ROS levels characteristic of melanoma [96]. In addition, strategies employing pH-labile bonds or enzyme-cleavable motifs offer further precision, enabling selective activation within the acidic and protease-rich TME while sparing normal tissues [97]. Collectively, these microenvironment-guided designs represent a promising direction for enhancing both the safety and therapeutic window of PROTAC-based interventions in melanoma.

In addition to molecular-level optimization, the use of targeted delivery platforms offers another effective avenue for mitigating off-target toxicity in melanoma. The high expression of certain surface markers, such as the melanocyte lineage markers gp100, Melan-A/MART-1, and the melanoma-specific chondroitin sulfate proteoglycan (MCSP), provides opportunities for receptor-mediated selective delivery [98]. A representative example is the folate-modified PROTAC folate–ARV-771, which leverages the overexpression of folate receptors on certain cancer cells to achieve preferential uptake and selective degradation of BRD family proteins in receptor-positive tumors, while largely sparing normal tissues with minimal folate receptor expression [99]. Building on this concept, future delivery strategies that target melanoma-associated surface markers, including MCSP or integrin subtypes, may further strengthen tumor accumulation of PROTACs, thereby improving therapeutic precision and reducing systemic exposure-related toxicity.

Taken together, these approaches underscore the value of designing PROTAC systems that are both selectively activated and efficiently delivered within the melanoma context by exploiting its distinctive microenvironmental characteristics. By aligning drug activation and transport with tumor-specific biological cues, these strategies offer a practical route to minimizing systemic toxicity while improving intratumoral efficacy. Ultimately, such integrated design principles are expected to play a pivotal role in accelerating the clinical translation of PROTAC-based therapies in melanoma.

### Off-target effects

Although PROTACs are often considered more selective than conventional small-molecule inhibitors, largely because their activity depends on the formation of a cooperative ternary complex, unintended off-target effects may still arise in certain biological contexts. This concern becomes especially pertinent in melanoma, where significant intratumoral heterogeneity and variable drug penetration generate pronounced spatial barriers to drug distribution that are distinct from those observed in other tumor types [100]. As a consequence, PROTAC exposure within the TME can be uneven, increasing the likelihood of aberrant target protein degradation not only in malignant cells but also in neighboring stromal and immune compartments [101].

Importantly, PROTAC selectivity is governed not only by the binding characteristics of the warhead toward the target protein, but also by the capacity of the system to assemble a stable, productive ternary complex between the protein of interest, the PROTAC molecule, and the recruited E3 ubiquitin ligase. In a seminal study, Bondeson et al. [102] reported that despite employing a promiscuous kinase inhibitor capable of binding over 50 kinases, the corresponding PROTAC selectively induced degradation of fewer than 15 targets. This discrepancy was attributed to the stringent structural and biophysical requirements for productive protein–protein interactions that stabilize ternary complex formation, thereby introducing an additional layer of selectivity beyond simple target engagement. However, this selectivity can be attenuated under non-ideal dosing conditions. At sufficiently high intracellular concentrations, PROTACs may exhibit reduced degradation efficiency and diminished specificity due to the so-called “Hook effect,” in which excessive compound levels disrupt optimal ternary complex assembly [103]. In melanoma, this limitation may be further intensified by the presence of highly heterogeneous vascular permeability and irregular intratumoral drug distribution, which can lead to local drug concentrations that are either suboptimal or supraoptimal for productive ternary complex formation. Under such conditions, PROTAC molecules may preferentially accumulate in stromal compartments and form non-productive binary complexes, rather than achieving optimal ternary complex assembly within malignant cells. These E3 ligase–associated binary interactions may inadvertently facilitate the recruitment and ubiquitination of off-target, low-affinity substrates, thereby increasing the likelihood of unintended protein degradation events [104]. In addition, emerging evidence indicates that certain CRBN-based PROTACs can trigger degradation of endogenous CRBN substrates, including IKZF1/3, ZNF827, and ZFP91 [105]. Among these, transcription factors such as IKZF1/3 are critical regulators of hematopoietic development and immune system homeostasis [106], suggesting that their unintended depletion could have clinically relevant consequences. This concern is particularly significant in melanoma, where profound immunosuppression within the TME is already a major obstacle to effective immunotherapeutic intervention, and inadvertent depletion of essential immune regulators could further compromise antitumor immunity [107].

To overcome these limitations, advanced proteomic approaches, particularly mass spectrometry based global degradation profiling, have become invaluable for systematically assessing the selectivity and safety landscape of PROTACs in melanoma models. Such techniques enable comprehensive mapping of degradation events across the proteome, thereby providing critical insight into both on-target activity and unintended substrate engagement. In parallel, strategies that enable external control over PROTAC activation have shown considerable promise in reducing off-target toxicity. By integrating stimuli-responsive modules into PROTAC architectures, their activity can be precisely regulated in both space and time through externally applied triggers, such as light [108–110]. For example, Deiters and colleagues [110] reported a photo-caged PROTAC targeting estrogen-related receptor α (ERRα), which remained pharmacologically inert until activated by UV irradiation. Although this system was initially demonstrated in breast cancer models, the underlying principle is readily adaptable to melanoma, where localized activation within tumor regions could substantially enhance tumor specificity while limiting systemic exposure, a particularly important consideration given the potential for severe immune-related adverse events in melanoma patients receiving combination immunotherapy. Collectively, these advances highlight the value of combining high-resolution proteomic evaluation with externally controllable activation strategies to improve the precision, safety, and translational potential of PROTAC-based therapies.

### Melanoma-specific challenges

Beyond the general limitations discussed above, several melanoma-specific challenges warrant particular attention, as they directly impact the translational feasibility of PROTAC-based strategies in this malignancy.

First, the potential interplay between PROTAC-mediated degradation and established BRAF/MEK inhibitor resistance pathways represents a critical yet underexplored issue. BRAF V600E mutations occur in approximately 40–50% of all melanomas, and BRAF inhibitors (vemurafenib, dabrafenib) combined with MEK inhibitors (trametinib) remain a standard-of-care for this patient subset [111]. However, acquired resistance to these targeted therapies inevitably develops through multiple mechanisms, including reactivation of MAPK signaling via NRAS mutations, MEK mutations, alternative splicing of BRAF V600E, and activation of bypass pathways such as PI3K-AKT [112]. Importantly, many of the proteins driving resistance—such as mutant BRAF, MEK, and components of the PI3K-AKT pathway—are themselves amenable to PROTAC-mediated degradation. This raises the intriguing possibility that PROTACs targeting these proteins could overcome resistance to conventional BRAF/MEK inhibitors. Conversely, chronic exposure to BRAF inhibitors has been shown to induce transcriptional and epigenetic reprogramming that may affect the expression of E3 ligases or components of the ubiquitin-proteasome system, potentially altering the sensitivity of melanoma cells to subsequent PROTAC treatment [112]. Furthermore, the high mutational burden and genomic instability characteristic of melanoma may accelerate the acquisition of resistance mechanisms to PROTACs themselves, including mutations in the target protein that abolish ligand binding or alterations in the E3 ligase pathway (e.g., CRBN or VHL mutations) [113]. To date, systematic investigations of these interactions remain scarce, and future studies should prioritize evaluating PROTAC efficacy in BRAF-mutant melanoma models with acquired resistance to BRAF/MEK inhibitors, as well as exploring rational combination or sequential therapy regimens.

Second, melanoma cells are known to exhibit high expression of ATP-binding cassette (ABC) efflux transporters, particularly ABCG2 (breast cancer resistance protein) and ABCB1 (P-glycoprotein) [114]. These transporters actively pump a wide range of chemotherapeutic agents and targeted therapeutics out of cells, contributing to multidrug resistance (MDR). PROTACs, given their relatively large molecular size (typically > 800 Da) and often hydrophobic or moderately lipophilic character, may serve as substrates for these efflux pumps, leading to reduced intracellular accumulation and diminished degradation efficacy [115]. Several studies have documented that resistance to BRAF and MEK inhibitors in melanoma is partly mediated by upregulation of ABCB1 and ABCG2 [114, 116], suggesting that PROTACs targeting key oncogenic drivers in melanoma may face similar efflux-mediated resistance mechanisms. Combinatorial approaches using efflux pump inhibitors, such as verapamil or tariquidar, have been proposed to enhance intracellular retention of therapeutic agents [117], although the potential toxicity and off-target effects of such combinations warrant careful evaluation. Alternatively, designing PROTACs with reduced susceptibility to efflux transport through strategic modification of physicochemical properties—such as reducing lipophilicity or optimizing polar surface area—represents a promising direction [118]. Additionally, the development of prodrug strategies that become active only upon intracellular conversion may help bypass efflux-mediated resistance.

Third, the degradation of transmembrane immune checkpoint proteins such as PD-L1 via traditional PROTACs poses a fundamental mechanistic challenge. Conventional PROTACs are designed to recruit the ubiquitin-proteasome system (UPS), which primarily targets cytosolic and nuclear proteins for degradation. PD-L1, however, is a type I transmembrane protein with its extracellular domain exposed to the tumor microenvironment and only a short cytoplasmic tail [119]. Following ubiquitination, transmembrane proteins are typically sorted to the lysosome rather than the proteasome for degradation [119]. This inherent mechanistic limitation explains why many reported PD-L1-targeting PROTACs exhibit only moderate degradation efficiency or require high concentrations to achieve meaningful effects. To address this challenge, researchers are developing novel induced proximity strategies that redirect transmembrane proteins to the lysosomal degradation pathway. For instance, lysosome-targeting chimeras (LYTACs) and antibody-based PROTACs (Ab-PROTACs) have been designed to exploit cell-surface receptors such as the asialoglycoprotein receptor (ASGPR) or the cation-independent mannose-6-phosphate receptor (CI-M6PR) to shuttle target proteins to the lysosome [120, 121]. Similarly, PD-L1-targeting bispecific antibodies that engage the lysosomal sorting pathway are under active investigation. These alternative degradation modalities, while promising, remain at an early stage of development, and their tumor specificity, pharmacokinetic profiles, and in vivo efficacy in melanoma models require systematic evaluation. Moreover, the selection of appropriate E3 ligases for transmembrane protein degradation and the optimization of linker chemistry to facilitate cell-surface engagement represent additional layers of complexity that must be addressed.

Taken together, these melanoma-specific challenges—interactions with BRAF/MEK inhibitor resistance, efflux transporter-mediated drug extrusion, and the intrinsic difficulty of degrading transmembrane proteins via the UPS—highlight the need for tailored PROTAC design strategies that account for the unique biological and pharmacological features of melanoma. Addressing these issues will require close collaboration between medicinal chemists, pharmacologists, and clinical oncologists to ensure that PROTAC-based approaches are optimally configured for this challenging malignancy.

## Conclusion and future perspectives

PROTAC technology has emerged as a transformative modality in cancer therapy, offering a mechanistic departure from traditional enzyme inhibition by enabling targeted degradation of oncogenic and immune-regulatory proteins. In the context of melanoma immunotherapy, PROTACs hold significant promise for overcoming resistance to immune checkpoint blockade. As summarized in this review, PROTACs can enhance tumor immunogenicity by restoring MHC-I antigen presentation, reshape the immunosuppressive tumor microenvironment through degradation of key regulators in Tregs, MDSCs, and TAMs, and directly suppress oncogenic signaling pathways that dampen antitumor immunity. Moreover, early preclinical evidence supports the combination of PROTACs with PD-1/PD-L1 or CTLA-4 blockade, suggesting synergistic potential.

Despite these advances, several critical challenges remain. Poor cell permeability, off-tissue toxicity, off-target effects, and the emergence of acquired resistance continue to hinder the clinical translation of PROTACs. Addressing these limitations will require a multidisciplinary effort. Future directions should prioritize: (1) the rational design of next-generation PROTACs with optimized physicochemical properties, including reduced molecular weight and improved membrane permeability; (2) the development of tumor microenvironment-responsive and tissue-specific delivery systems, such as nanocarriers, antibody-PROTAC conjugates, and prodrug strategies, to minimize systemic toxicity; (3) the application of advanced proteomic and CRISPR-based screening platforms to systematically evaluate off-target degradation profiles and identify resistance mechanisms; and (4) the design of partial or context-dependent degraders, particularly for transcription factors with pleiotropic functions in T cell biology.

Furthermore, the immunomodulatory effects of PROTACs remain underexplored. Most current studies focus on tumor cell-intrinsic outcomes, whereas the direct impact of PROTACs on immune cell subsets—such as CD8⁺ T cell exhaustion, dendritic cell maturation, and B cell function—warrants systematic investigation. Combining PROTACs with other immunomodulatory strategies, including oncolytic viruses, cytokines, or epigenetic modulators, may further broaden their therapeutic utility.

In conclusion, PROTAC technology represents a powerful and versatile platform to sensitize melanoma to immunotherapy. While significant hurdles remain, continued innovation in chemical biology, drug delivery, and immuno-oncology will be essential to unlock the full potential of PROTAC-based combination regimens for melanoma patients.

## Data Availability

No datasets were generated or analysed during the current study.
